# Drug pricing reform in China: analysis of piloted approaches and potential impact of the reform

**DOI:** 10.3402/jmahp.v4.30458

**Published:** 2016-03-15

**Authors:** Yixi Chen, Shanlian Hu, Peng Dong, Åsa Kornfeld, Patrycja Jaros, Jing Yan, Fangfang Ma, Mondher Toumi

**Affiliations:** 1 Pfizer Investment Co. Ltd, Beijing, China; 2 Shanghai Health Development Research Center, Shanghai, China; 3 Creativ-Ceutical, Paris, France; 4 Creativ-Ceutical, Krakow, Poland; 5 Creativ-Ceutical, London, UK; 6 Creativ-Ceutical, Beijing, China; 7 Faculty of Medicine, Department of Public Health, Aix-Marseille University, Marseille, France

**Keywords:** China, drug pricing, healthcare reform, reference pricing

## Abstract

**Objectives:**

In 2009, the Chinese government launched a national healthcare reform programme aiming to control healthcare expenditure and increase the quality of care. As part of this programme, a new drug pricing reform was initiated on 1 June 2015. The objective of this study was to describe the changing landscape of drug pricing policy in China and analyse the potential impact of the reform.

**Methods:**

The authors conducted thorough research on the drug pricing reform using three Chinese databases (CNKI, Wanfang, and Weipu), Chinese health authority websites, relevant press releases, and pharmaceutical blogs and discussion forums. This research was complemented with qualitative research based on targeted interviews with key Chinese opinion leaders representing the authorities’ and prescribers’ perspectives.

**Results:**

With the current reform, the government has attempted to replace its direct control over the prices of reimbursable drugs with indirect, incentive-driven influence. Although the exact implementation of the reform remains unclear at the moment, the changes introduced so far and the pilot project designs indicate that China is considering adaptation of some form of internal and external reference pricing policies, commonly used in the Organisation for Economic Co-operation and Development countries. Several challenges related to the potential new mechanism were identified: 1) the risk of hospital underfunding, if hospital funding reform is not prioritised; 2) the risk of promoting the use of cheap, low-quality drugs, if a reliable quality control system is not in place and discrepancy between the available drugs is present; 3) the risk of increasing disparity in access to care between poor and rich regions, in case of country-wide price convergence; and 4) the risk of industry underinvestment, resulting in reduced competition, issues with quality and sustainability of supply, and potentially negative social impact.

**Conclusions:**

Foreign pricing policies cannot be transferred to China without prioritising historical, cultural, and economic contextualisation. Otherwise, the new policy may be counterproductive and affect the whole healthcare chain, as well as the health outcomes of Chinese patients.

Over the last years, the government of China has been continuously tackling the issue of growing healthcare expenditure. Between the years 2000 and 2013, overall spending on healthcare increased almost sevenfold, from approximately RMB 459 billion (USD 75 billion at the current exchange rate^1^) to RMB 3,167 billion (USD 519 billion) (1). It is projected to reach USD 1 trillion in 2020 (2). Total *per capita* expenditure rose from RMB 358 (USD 59) in 2000 to RMB 2,286 (USD 375) in 2013, with an average annual growth of approximately 15.4%. At the same time, total healthcare spending as a proportion of gross domestic product (GDP) increased by only one percentage point – from 4.6 to 5.6% (0.07% per year on average) (1), while annual GDP growth remained at a high level of 10% on average (3). With significant extension of basic insurance programmes over last several years and more than 95% of the Chinese population currently entitled to some form of public coverage (4), the makeup of these expenditures changed substantially. Contributions from public sources – namely from government and social health – increased from 46% in 2000 to 62% in 2011. Chinese patients paid the remaining 38% out of pocket (5).

The high costs of healthcare may be partly explained by the surge in pharmaceutical costs. Between the years 2000 and 2009, China observed an average annual growth rate in real *per capita* pharmaceutical spending of 9.6%. In 2009, pharmaceutical expenses constituted 42.5% of total healthcare expenditure, compared with an average of 15.6% in countries belonging to the Organisation for Economic Co-operation and Development (OECD). For other Asian countries, like Japan and South Korea, the relevant numbers were also substantially lower – 20.6 and 25.1%, respectively (6). This high proportion may be partly explained by the fact that the healthcare costs are mainly driven by manpower, which is very cheap in China. In addition, Chinese doctors are allowed to dispense drugs, which constitute an additional source of income for them and create incentives for overprescription (7). Over a period of 5 years, the market value of Western pharmaceuticals in China more than doubled, from USD 27 billion in 2006 to USD 71 billion in 2011 (in ex-factory prices). A similar pace of growth – from USD 6 billion to 13 billion – was observed at the same time for the traditional Chinese medicine market (2).

Despite growing healthcare expenditures and the increasing contribution of public funds to overall healthcare spending, the level of satisfaction with the quality of care remains low among Chinese patients (8). Moreover, significant inequity exists across the country in terms of accessibility, treatment outcomes, and the related financial burden: between the provinces, urban and rural populations, and different income groups (9). As an example, for the urban population the out-of-pocket payment as a share of average annual household living consumption expenditure increased from 6.4% in 2000 to 7.6% in 2005 and then gradually decreased to 6.4% in 2012. For the rural population it continued to increase from 5.2% in 2000 to 8.7% in 2012 (10).

To address growing healthcare expenditure accompanied by the issue of quality and accessibility to care, in 2009 the Government of China launched a reform programme under which a universal healthcare system is to be established by 2020. The programme targets different healthcare sectors and aims to provide all citizens with affordable and quality healthcare covered by health insurance (11). Progress has been already made with respect to several key areas such as enrolment in insurance schemes, development of community health infrastructure in urban and rural areas, and improvement in access to essential medicines (12).

Since pharmaceutical sales constitute a significant part of the total healthcare expenditure, the reform programme also aims to introduce a new pricing mechanism allowing more efficient utilisation of the national pharmaceutical budget. A new drug pricing reform (‘Opinions on Promoting the Drug Pricing Reform’ [2015] No. 904) (13) initiated on 1 June 2015 seems to have been inspired by internal (IRP)^2^ and external reference pricing (ERP)^3^ policies, which are commonly used as an effective pharmaceutical cost-containment tool in OECD countries. In China, manufacturers are free to set market prices, unless the drug is included in the Health Insurance Formulary (HIF)^4^ or other government-subsidised programme. Until 1 June 2015 the government directly participated in the pricing of reimbursable drugs. The National Development and Reform Commission (NDRC) set exact retail prices for selected drugs, such as those provided under government programmes of planned supply (so-called government pricing or GP) and maximum retail prices or price caps for the remaining reimbursable drugs (so-called government-guided pricing or GGP) (20). Prices were set for each active ingredient and dosage form and were derived mainly from the manufacturers’ costs (development, manufacturing, etc.). Local governments could adjust the maximum prices. Some products, considered to have higher quality than their generic forms, were permitted to have prices higher than the maximum retail prices indicated by GGP (i.e. ‘individual’ pricing or pricing ‘privilege’). Actual retail prices were set through local tenders. Selected manufacturers were allowed to provide their products to hospitals at prices established through a bidding process (i.e. local procurement prices). There was no standardised reimbursement price – drugs were reimbursed based on their actual price (4). The drug pricing reform aims to introduce changes to this paradigm. It is supported by several pilot programmes. Two of them – in the cities of Sanming and Shaoxing – were initiated before the reform and were still in operation, and another one – in the municipality of Chongqing – was about to commence at the time of writing. They aim to test various pricing approaches and their outcomes will likely impact future pricing algorithms.

The objective of this article was to describe the changing landscape of the drug pricing policy in China, including future trends. In addition, this study aimed to analyse the potential impact of the reform on various healthcare stakeholders, essentially on patients and their access to pharmaceuticals, and put it in the context of international experience with reference pricing.

## Materials and methods

As the first step, we conducted thorough research aiming to collect all publicly available information related to the current Chinese drug pricing reform and pilot programmes. We used the keywords ‘pricing’, ‘reimbursement’, ‘policy’, and ‘reform’ to search the three main Chinese databases: China National Knowledge Infrastructure (www.cnki.net), Wanfang Data (www.wanfangdata.com.cn), and Weipu (www.cqvip.com). In order to retrieve only the most recent and relevant articles, we applied a time limit of 1 January 2012 and used additional keywords limiting the results to China-related articles (see Supplementary Table 2). The database search was performed on 17 April 2015. In addition to the databases, we searched Internet resources: Chinese government and healthcare authority websites (NDRC: www.ndrc.gov.cn; National Health and Family Planning Commission: www.nhfpc.gov.cn; Ministry of Human Resources and Social Security: www.mohrss.gov.cn for national-level information; and the corresponding local-level authority websites), the grey literature, relevant press releases, blogs, and common pharmaceutical discussion forums. An additional search was concluded by the end of April 2015. We developed an extraction table to ensure all relevant information was organised in a format that was easy to access and analyse. For redundant information we saved the most reliable source, applying the following ranking for reliability of sources: healthcare authority website, scientific article, official newspaper, press release, blog, discussion forum. Information was consolidated and reported initially as a PowerPoint presentation.

As the next step, we conducted primary research, namely targeted interviews with Chinese key opinion leaders (KOLs) representing either the authorities’ or prescribers’ perspectives. The interviews aimed to fill the information gaps after the secondary research and to understand appreciation of the current pricing reform among relevant stakeholders. We designed two questionnaires: one for the authority representatives and another for prescribers. Both questionnaires contained three main sections aiming to do as follows: 1) obtain interviewees’ insights on the general environment of the reform; 2) validate information collected through the secondary research and fill information gaps; and 3) obtain interviewees’ opinions on the future drug pricing approach. The authorities’ questionnaire contained, in addition, a section aiming to collect interviewees’ insights on the international experience in drug pricing and its potential applicability to China. We interviewed nine KOLs in total: six authority representatives and three prescribers (see Supplementary Table 3 for the interviewees’ profiles). All interviews took place between 20 and 25 May 2015. They were conducted in a one-on-one fashion, during face-to-face meetings, or over the phone and lasted on average 1 hour each. The interviewees’ answers were combined with the results of the secondary research in order to provide the most comprehensive overview of the current situation in China and an appreciation of the forthcoming changes.

Finally, at the stage of preparation of the manuscript, additional interviews were performed to clarify all outstanding issues, and secondary research was updated on 26 June 2015. The results were split between the national pricing reform concept and pilot programmes that were already implemented or announced.

## Results

### The Chinese experience with drug pricing and the changes introduced on 1 June 2015

At the beginning of May 2015, the NDRC announced implementation of changes to the current pricing model with effect from 1 June 2015 (13). The core component of the reform was abolishment of GP and GGP for most drugs and introduction of new mechanisms of price control (Table 1). The NDRC announced the introduction of a ‘reimbursement standard’ acting as a guide for the market prices of drugs included in the HIF for which there is an existing market competition and understood as a form of a reference price (or reimbursement level) used in IRP systems. The Ministry of Human Resources and Social Security was assigned a leading role in this process. However, no definition of *reimbursement standard* was provided in the announcement and, at the time of writing, Chinese local administrative divisions had autonomy to apply their own methodology before introducing final regulations at the national level (21). *Reimbursement standard* will not be used for HIF drugs with little or no market competition (i.e. in-patent drugs, exclusively produced traditional Chinese medicines). For these products retail prices will be established by multilateral negotiations involving the pharmaceutical industry and other stakeholders. For blood products not included in the HIF and drugs procured and subsidised by the government (i.e. vaccines, HIV/AIDS drugs, contraceptives), retail prices will be generated either through tenders or established by negotiations. However, the announcement did not specify the framework for price negotiations. The local tendering system was not abolished by the new law. Therefore, at least at the moment, the prices of most drugs are still affected by the competitive local bidding process.

**Table 1:** New pricing mechanisms introduced on 1 June 2015 (13)

Group of products	New pricing mechanism
Drugs included in the HIF with existing market competition^1^	Reimbursement standard
Drugs included in the HIF with little or no market competition	Multilateral negotiations
(i.e. in-patent drugs, exclusively produced traditional Chinese medicines)^1^	
Blood products and drugs procured and subsidised by the government	Tenders or multilateral negotiations
(i.e. vaccines, HIV/AIDS drugs, contraceptives)^1^,^2^	

HIF: Health Insurance Formulary.

1 Except Class I psychotropics and anaesthetics, which remained under the government guided pricing.

2 Applies to blood products not included in the HIF.

### Pilot projects

China is currently running several pilot projects to inform the implementation of the drug pricing reform.

#### Sanming

Sanming – a prefecture-level city in Fujian Province (South of China) – is piloting a form of IRP for drugs with the same active ingredient and dosage form. The reimbursement standard is set at the procurement price of the cheapest generic from the group and patients are liable to cover an excess amount in addition to any other applicable co-payment (22).

The pilot was introduced on 1 June 2014 and follows a series of earlier local policy changes aiming at increasing the use of generics, reducing malpractice and overprescription, and optimising the use of the healthcare budget. It was initiated in 22 public hospitals and applied to 16 top-prescribed molecules and dosage forms. According to the initial announcement, the selected drugs were supposed to display ‘a minimal difference in terms of quality and a big difference in terms of price’ (22). The above criteria were not explicitly defined in the announcement. However, in practice, each IRP group must have contained both an imported drug (i.e. off-patent originator) and locally produced generics.

The prices of drugs in Sanming were reported to decrease significantly. As an example, in February 2015 the cost of the anti-breast cancer drug exemestane was reported to be five times lower as compared with the pre-pilot level (from RMB 657 to 136, i.e. from USD 107.7 to 22.3 per box^5^). The price of the proton pump inhibitor omeprazole fell more than 30 times (from RMB 256 to 7.8, i.e. from USD 42 to 1.3 per box^5^) (23). This was probably a result of changing preference towards cheaper, locally produced generics. Based on information collected through the primary research, the overall purchase value of selected imported drugs dropped down by approximately 12 to 54% (Table 2). Some higher priced products disappeared from the market. Among all molecules with a monthly purchase value over RMB 200,000, the value market share of products from Chinese manufacturers increased by 14.4 percentage points between August 2014 and March 2015 (from 44.4 to 58.8%), which suggests that locally produced generics took the place of imported drugs. The government of Sanming aims to continue with the pilot and increase the number of involved molecules.

**Table 2:** Changes in the purchase value of selected imported drugs (Sanming)

			Change in market share from February 2015 to March 2015^1^ (by value)		
			
Active ingredient	Brand; manufacturer	Dosage form	Feb 2015 [USD]	Mar 2015 [USD]	Δ
Amlodipine	Norvasc; Pfizer	5 mg×7 tab	45,910.00	40,345.20	−12.1%
Cefoperazone/Sulbactam	Sulperazon; Pfizer	1500 mg×1 vial	52,635.10	44,841.50	−14.8%
Aspirin	Aspirin; Bayer	100 mg×30 tab	23,987.40	19,630.00	−18.2%
Acarbose	Glucobay; Bayer	50 mg×30 tab	68,822.60	55,119.00	−19.9%
Cefuroxime	Monocef; Esseti Farmaceutici	1500 mg×1 vial	978.20	677.20	−30.8%
Atorvastatin	Lipitor; Pfizer	20 mg×7 tab	79,886.50	36,573.30	−54.2%

1 The reported period is the one for which data were collected through the primary research; data for the overall piloting period or other time spans were not available.

#### Chongqing

Chongqing – the municipality directly under the Central Government (Southwest of China) – announced the initiation of the drug pricing pilot project on 1 January 2015 (24). However, as of June 2015, the pilot was still not in operation. It was planned to incorporate all municipal public hospitals and applied to the 300 top-prescribed molecules and dosage forms (24).

**Fig. 1.**
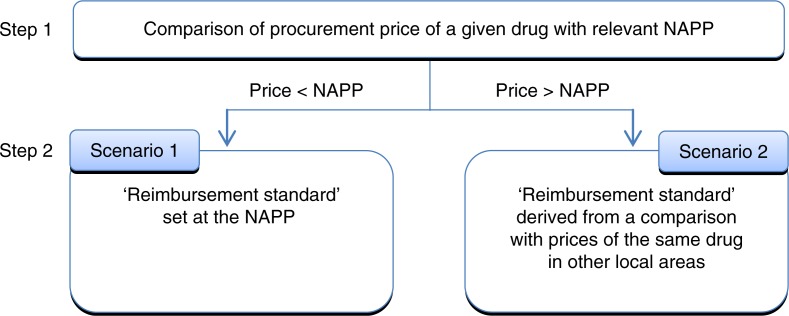
Overview of Chongqing pricing methodology (24). NAPP: national average procurement price.

Different piloting approaches have been under discussion since the time of announcement (24, 25). Based on the most recent information (June 2015), in order to establish the reimbursement standard, Chongqing plans to use both elements of IRP and comparison with procurement prices of the same drug (i.e. a product from the same manufacturer) in other local areas (provinces, municipalities). As the first step, the procurement price of a given drug is to be compared with the national average procurement price (NAPP). The NAPP is calculated based on the previous year's procurement prices for all drugs across China with the same active ingredient and dosage form, except drugs that were under ‘individual’ pricing before 1 June 2015. In addition, the products included in the calculation must meet the national GMP criteria. Based on the results of this comparison, as the next step the reimbursement standard will be calculated according to two different algorithms (Fig. 1). If the procurement price of a given drug is lower than the relevant NAPP, the reimbursement standard will be set at the NAPP (Scenario 1). If the procurement price is higher, the reimbursement standard will be derived from a comparison with the prices of exactly the same product in other local areas (Scenario 2). Table 3 indicates the exact methodology. Scenario 2 bears some characteristics of ERP; however, in this case, the comparison does not involve foreign countries but other relatively autonomous areas within the same country.

**Table 3:** Reimbursement standard calculation methodology for drugs for which the procurement price is higher than the NAPP (Chongqing) (24)

Number of available procurement prices from other local areas^1^	Methodology for calculation of reimbursement standard
≥3	The average of the three lowest provincial procurement prices of this drug
2	The lower of two provincial procurement prices of this drug
1	Certain %^2^ below the provincial procurement price of this drug

NAPP: national average procurement price.

1 Number of procurement prices available from other local areas from the previous year.

2 % depends on last year's rank on the Chongqing's best sellers list: 5% if top 100; 4% if within 101–200; and 3% if within 201–300.

It is expected that most locally produced generics, which are normally much cheaper than imported off-patent originals, will enter Scenario 1. It was not explicitly stated in the announcement, but it seems that the difference between the actual procurement price and the reimbursement price will constitute the hospital's profit. The patients’ co-payment will be the same regardless of the product they are prescribed, because it will be derived from the relevant NAPP. Imported drugs and drugs previously under individual pricing will more likely enter Scenario 2 (24). If their procurement price is higher than the applicable reimbursement standard, both patient and hospital will be liable to cover the excess amount (30 and 70%, respectively, on average).

The methodology of the Chongqing pilot project is perceived as complicated and sensitive to the potential lack of pricing data from other local areas, which is required for calculation. The impact upon its actual implementation is still yet to be seen.

#### Shaoxing and others

Shaoxing – a prefecture-level city in Zhejiang Province (Northeast of China) – is piloting an approach called ‘second price negotiation’, which allows hospitals to negotiate discounts directly with suppliers (26). Local procurement prices obtained via the tendering process (here interpreted as the ‘first price negotiation’) serve as both price caps in negotiations and reimbursement standards. Hospitals hand out obtained discounts to the provincial Bureau of Finance. This money is to be further invested in hospitals.

The pilot was introduced on 1 January 2015 in all-level public hospitals and applied to all drugs purchased through the provincial procurement platform. As reported at the end of January 2015, the prices of most drugs’ in Shaoxing had been discounted by approximately 4 to 10% (27). Higher discounts were applied to generics as compared with off-patent originals, as well as to products purchased in greater amounts (Table 4) (27). Some high priced products were reported to be withdrawn from the local market. For instance, Shaoxing 7^th^ People's Hospital discontinued or limited the use of 14 drugs, most of them being imported products from multinational companies (e.g. Pfizer's cefoperazone/sulbactam, Wyeth's venlafaxine, Lundbeck's escitalopram; see Supplementary Table 4 for the full list) (27).

**Table 4:** Change in the prices of the 200 top-prescribed drugs due to second price negotiation during the first month of implementation (Shaoxing) (46)

	Change in prices of 200 top-prescribed drugs during Jan 2015		
	
Type of products	Drugs ranked 1–50	Drugs ranked 51–100	Drugs ranked 101–200
Generics	−10%	−8%	−6%
Off-patent originals	−5%	−2%	−2%

Shaoxing is not the only city piloting the second price negotiation. The province of Anhui initiated a similar project in March 2015, with the difference that the discounts obtained through negotiation are to be awarded directly to the involved hospitals (28). Until June 2015, there were in total 100 cities involved in the general healthcare reform that were allowed to pilot the second price negotiation (see Supplementary Table 5 for the full list) (29–31).

### Feedback from KOLs

The change in the pricing paradigm was perceived by the interviewed KOLs as a step towards a market economy and a more efficient healthcare system; however, they reported several challenges and concerns:

- The reform lacks systemic design and adequate supporting policies.
- Clear regulations for introduced changes and coordination mechanisms between responsible bodies are not established.
- The quality of generic drugs is not ensured.
- The Chinese pharmaceutical market in China is not mature.

Appreciation of different piloting approaches varied. In general, the Chongqing approach was more widely supported and considered suitable for China countrywide; however the methodology for the reimbursement standard calculation was considered too complicated and potentially limited by lack of data from other local areas required for calculation. Setting the reimbursement standard at the average rather than at the lowest procurement prices was considered more appropriate. In addition, in contrast with Sanming, the Chongqing approach was perceived as taking into account differences in drug quality and price levels (i.e. the ‘individual’ approach to higher priced drugs, avoiding the risk that these drugs may step out of the market). The second price negotiation was considered an approach that can temporarily decrease drug prices but can be harmful in the long term as it may generate the risk of corruption.

When asked about their insights on the international experience in drug pricing and its potential applicability to China, KOLs judged that ‘setting IRP groups for drugs with the same active ingredient and setting the reference price at the average price of drugs from the group’ was the most applicable and relevant approach for the Chinese market. Some of them were also in favour of setting an average price at ‘weighted average’. Most of the KOLs expressed that ‘using ERP to set prices of reimbursable drugs not included into the internal reference price group system’ was relevant and applicable to China (see Supplementary Table 6). The interviewees did not indicate any specific country that China could or should follow.

## Discussion

### Potential ‘shape’ of the reform

With the current reform, the Chinese government attempts to replace its direct control over the retail prices of reimbursable drugs with a more indirect influence. The abolishment of GP and GGP for most pharmaceuticals gives manufacturers, in theory, more freedom to set retail prices. However, in practice, prices are expected to continue to be constrained mainly by tenders and other factors, such as hospital budgets and controlled reimbursement levels. To maintain an indirect influence over drug prices, the government seems to consider adaptation of some form of the IRP or ERP policies commonly used in the OECD zone. Implementation of the reimbursement standard as a tool to guide the market prices of drugs included in the HIF clearly resembles IRP policies successfully practised in Germany, the Netherlands, Denmark, and many other foreign countries. Comparison with prices of the same drug in other local areas (e.g. provinces, municipalities) bears some characteristics of ERP. However, in this case, the comparison does not involve foreign countries, as per the definition, but other big and relatively autonomous areas within the same country. The role of the second price negotiation in the overall process remains unclear at the moment; specifically, it is unknown if it could be considered as the only pricing tool or if it would be used in conjunction with other mechanisms, like IRP or ERP.

Many elements of the new drug pricing reform remain unknown and will likely depend on the pilot project outcomes. Nonetheless, based on current knowledge and our understanding of the government's main assumptions underlying the reform, we undertake an attempt to predict and discuss its potential impact in terms of challenges and consequences.

### Main challenges and consequences

An important characteristic of the Chinese system is that most healthcare utilisation – in some cases even 80% – occurs at the hospital level (32). Moreover, hospitals hold a dual role: they are not only the main service provider but also the main supplier of pharmaceutical products. It is estimated that only approximately 18% of all prescription drugs (by volume) are sold via retail pharmacies (4). Even more relevant is that since the early 1980s dispensing drugs constituted an important source of financing for hospitals (33); hospitals were allowed to mark up drug prices by up to 15%. This created problematic incentives by encouraging overprescription and a preference towards more expensive products (34). In some cases, pharmaceutical sales were reported to constitute as much as 50 or even 90% of the overall hospital revenue and almost all of the profit (35). As a part of the general healthcare system reform, a zero mark-up policy for essential medicines was introduced in primary hospitals. This has led to a serious drop in hospitals’ income. Revenue loss was supposed to be offset by increased insurance and government subsidies; however, the compensation mechanisms were reported to be largely ineffective (35, 36). Comprehensive hospital financing reform, aiming at reducing the reliance of hospitals on drug sales as a major source of revenue, still remains ahead and its final shape is not known at the moment. By introducing the reimbursement standard for all HIF products, the drug pricing reform intends to motivate hospitals to sell lower priced drugs and to be more efficient in managing pharmaceutical expenses. The need for alternating incentives is unquestionable and desirable. It would benefit both payers and patients; however, within current settings, it puts successful implementation of the reform at risk. Hospitals will face further income loss, which, as in the case of income lost due to zero mark-up on essential drugs, may not be offset by increased provision of public funds (e.g. increased reimbursement for medical services or other forms of subsidies). This might be of particular issue especially in poorer regions. Under financial pressure, hospitals may increase user fees and/or reduce operating costs, resulting in lowering the standard of services provided, which would affect accessibility and/or quality of care. Both outcomes are contradictory to the overall goal of the Chinese healthcare reform. The KOLs interviewed expressed concern that, at the moment, the drug pricing reform lacks systematism. Improvement of other healthcare segments, in particular hospital funding and management, should be prioritised before changes to the drug pricing system. Otherwise there is a risk of hospital underfunding and negative impacts on health outcomes.

In contrast, second price negotiation seems to be contradictory to the goal of reducing reliance on drug sales and reinforcing the non-profit nature of public hospitals. It may be seen as an alternative way to generate revenue after introduction of the zero mark-up policy, providing hospitals are directly granted the discounted amount.

Another important issue in China is the therapeutic interchangeability of drugs (37–39). The KOLs interviewed consistently agreed that there are discrepancies in terms of quality between imported off-patent originals and some locally produced generics, whereas the therapeutic interchangeability of drugs and the capability to measure them properly are prerequisites for an effective and sustainable IRP system. Countries applying IRP policies have adequate instruments in place, assuring high quality and therapeutic equivalence of products placed in the same basket – robust bioequivalence studies for generics and health technology assessment for different molecules. Currently China does not have such instruments in place. Dissolution testing is accepted for approvals (40) and generic products can be used as comparators when an originator product is not available (41). The quality assurance system is still under development (42). In spite of this, based on the Sanming pilot, it seems that the government may assume that all drugs with the same active ingredient available on the Chinese market are equivalent and display the same safety and efficacy. This assumption is obviously questionable. Introducing the IRP system in such a context may pose several risks. On the one hand, it will promote the use of cheaper drugs, regardless of their quality. The potential use of low quality drugs will affect patients’ safety and treatment outcomes, if they are not fully bioequivalent to original drug. It will lead to a waste of resources on potentially harmful products and an undesired increase of expenditures in other healthcare sectors, resulting from development of side effects and disease complications – both preventable if adequate treatment is applied. It could also lead to an increase in the already significant inequity in access to healthcare between different income groups, if – as a result of limited reimbursement – only the wealthiest could afford high-quality drugs. In contrast, even if the IRP system was introduced only for drugs with proven quality, the low level of social trust in locally produced generics may paradoxically prevent successful implementation of the reform. Concerns about the quality of domestic drugs could have several undesired effects: 1) some patients may resign from treatment, if they are not willing to switch to generic and the co-payment amount for the off-patent original is too high; 2) for the same reason some patients may prefer to purchase only a part of the treatment course of the off-patent original (an amount they can afford) rather than a full-treatment course of a generic; 3) some patients may try to obtain off-patent originals from alternative, uncontrolled sources. All these risks could negatively impact patients’ health outcomes.

The Chongqing pilot suggests that the new pricing strategy may involve a comparison with other local areas to set or adjust the reimbursement standard. Given the size and administrative structure of China, this approach bears some characteristics of ERP policies practised by many foreign countries, although the comparison is being made within the same country. It has been suggested that ERP, through its spillover and circular (re-referencing) effect, leads to convergence of prices across countries (15, 18, 19). A similar effect, resulting in a uniform price, should be expected if cross-referencing between provinces (or other local areas) is introduced in China. This result may not be beneficial, considering that China is a country characterised by significant economic disparities and a strong correlation exists between provincial wealth and spending on medicine (41). Comparison with other local areas already influences prices; however, it does not take a form of a rigid requirement. Therefore, suppliers are able to acknowledge and appreciate differences in purchasing power and offer lower prices in low revenue areas, which they would not be willing to do at the overall country level. Implementation of strict cross-referencing rules will prevent suppliers from offering such discounts. To avoid downward price convergence they will aim to maintain similar prices across the country. This will impose a significant financial burden on already disadvantaged areas and/or may leave the poorest unsupplied, stimulating further increase in inequity to healthcare access. Non-uniform, differential intracountry tiered pricing has actually been proposed as an approach that could significantly increase healthcare access and affordability in countries like China (43).

Finally, IRP policies are recognised as effective cost-containment tools, reinforcing price competition and favouring generic penetration (15–17). This is true in mature markets. In China, where authorities may place the strongest attention on price and pay less attention to medicine quality and reliance of supplier performance, an excessive pursuit of lower prices may pose two important risks: 1) potential quality risks if, in order to ensure a satisfactory profit margin, the manufacturers decide to save on production costs; 2) manufacturers and/or suppliers stepping out of the market, resulting in no real price competition or even the disappearance of certain molecules from the market. For example, drug shortages have already been reported in the two pilot cities, Sanming and Shaoxing (44, 45). Lowering prices too much will certainly affect pharmaceutical companies. If they have no time to adjust to the upcoming changes and their revenue is significantly affected, they might be 1) discouraged from undertaking new investments and 2) forced to drastically adjust their business models by, for example, reducing current investment and potentially employment.

The results of this study should be interpreted in the context of its limitations. The current situation in China is very dynamic. Limited information is available. The universal healthcare system reform initiated in 2009 targets different sectors, and changes to the pricing paradigm are one of many. This adds complexity to the overall situation. Therefore, the pricing reform should be seen as an evolving process and its future ‘shape’ is to be further investigated.

## Conclusions

Although many elements remain unknown and will likely depend on the pilot project outcomes, we are already able to identify the critical assumptions underlying the reform: creating incentives for hospitals to be more efficient in managing drug expenses and treating all products with the same active ingredient as equivalent in terms of quality, efficacy, and safety. However, with hospitals economically depending on profit generated from drug sales and drug quality issues, these key foundations of the reform may distort the piloting exercise and lead to biased conclusions. The risk of hospitals underfunding does not seem to be adequately balanced and the quality assurance system is still under development despite the fact that it has been improving. Moreover, comparison with other local areas, understood as a form of external reference pricing, may have potentially deleterious consequences on supply and equity towards poorest regions. Abolishment of GP and GGP, as such, is not expected to affect drug prices, as other mechanisms of price control remain in place. Some elements of the reform seem not to be well aligned or even contradictory, like introduction of the reimbursement standard and maintaining the tendering system. This indicates that, given complexity of the market, foreign pricing policies cannot be transferred to China without being properly adjusted for local healthcare specificities.

A comprehensive and consistent reform that integrates the full chain of healthcare services delivery, including pharmaceuticals, is required. Finally, the current hospital-centric system should evolve towards a more patient-centric approach. This will likely lead to a reduction of the hospital role in the overall system compensated by a light, decentralised, agile and effective ambulatory care unit and office-based medical practice. Such change would significantly reduce management costs and allow allocation of the current heavy administration cost to healthcare services and pharmaceuticals.

## Supplementary Material

Drug pricing reform in China: analysis of piloted approaches and potential impact of the reform

## Footnotes

1 Assumed exchange rate: USD 1=RMB 6.1

2 *IRP* refers to ‘the practice of using the price(s) of identical medicines (ATC 5 level) or similar products (ATC 4 level) or even with therapeutic equivalent treatment (not necessarily a medicine) in a country in order to derive a benchmark or reference price for the purposes of setting or negotiating the price or reimbursement of the product in a given country’ (14). IRP aims to limit expenditure on the reimbursement of pharmaceuticals by making use of the existence of equivalent products on the national market and setting a reference price (or reimbursement level) for a group of drugs that are considered to be therapeutically interchangeable. It reinforces price competition and favours generic penetration. Methodologies for assuming therapeutic interchangeability of drugs and reference price calculation vary between countries (Supplementary Table 1) (15–17).

3 *ERP* refers to ‘the practice of using the price(s) of a medicine in one or several countries in order to derive a benchmark or reference price for the purposes of setting or negotiating the price of the product in a given country’ (14). It is widely used by OECD countries as a main or supportive pricing criterion, mainly to control the prices of reimbursable pharmaceuticals that are protected by intellectual property rights and benefit from a legal monopoly. It is considered to be an effective and easy-to-implement cost-containment tool leading to lowered prices of in-patent drugs (Supplementary Table 1) (15, 18, 19).

4 The Health Insurance Formulary includes the National Reimbursement Drug List and Provincial Reimbursement Drug List.

5 Exact dose and pack size not reported.
