# Supplementary material for: Drug pricing reform in China: analysis of piloted approaches and potential impact of the reform
Source: J Mark Access Health Policy. 2016 Mar 15;4:10.3402/jmahp.v4.30458. doi: 10.3402/jmahp.v4.30458 (PMC4802695; doi:10.3402/jmahp.v4.30458)
Supplement: Drug pricing reform in China: analysis of piloted approaches and potential impact of the reform [file JMAHP-4-30458-s001.pdf]

Supplementary files

Table S1. The use of different IRP & ERP policies in selected European countries (15, 16)

	**Example approaches**	**BE**	**DE**	**DK**	**NL**	**FR**
**IRP**	Active ingredient-based internal reference price groups	X	X	X	X	X
	Pharmacological class-based internal reference price groups		X		X	
	Therapeutic class-based internal reference price groups		X		X	
	Reference price at the average price of drugs from the group					X
	Reference price at the weighted average price of drugs from the group		X[1]		X[2]	
	Reference price at the fixed % below the price of the off-patent original drug	X				
	Reference price at the cheapest drug from the group			X		
**ERP**	ERP to set or adjust prices of reimbursable drugs outside the IRP system	X	X[3]	X	X	X
	ERP to set or adjust prices of reimbursable drugs inside the IRP system				X	

BE – Belgium, DE – Germany, DK – Denmark, ERP – external reference pricing, IRP – internal reference pricing, NL – the Netherlands, FR – France

1 – Reference price calculated by regression analysis (econometric model)

2 – Reference price set directly below the weighted average price of medicines

3 – For new hospital-only medicines

Table S2. Search strategy

**China National Knowledge Infrastructure:** http://www.cnki.net	
Search 1	(SU=('医保支付') OR SU=('药品支付') OR SU=('药品参考价') OR SU=('药品参考定价') OR SU=('药价改革') OR SU=('药品定价改革') OR TI=('医保支付') OR TI=('药品支付') OR TI=('药品参考价') OR TI=('药品参考定价') OR TI=('药价改革') OR TI=('药品定价改革') OR KY=('医保支付') OR KY=('药品支付') OR KY=('药品参考价') OR KY=('药品参考定价') OR KY=('药价改革') OR KY=('药品定价改革') OR AB=('医保支付') OR AB=('药品支付') OR AB=('药品参考价') OR AB=('药品参考定价') OR AB=('药价改革') OR AB=('药品定价改革')) AND (FT=('德国') OR FT=('台湾') OR FT=('日本'))
Search 2	(SU=('医保支付') OR SU=('药品支付') OR SU=('药品参考价') OR SU=('药品参考定价') OR SU=('药价改革') OR SU=('药品定价改革') OR TI=('医保支付') OR TI=('药品支付') OR TI=('药品参考价') OR TI=('药品参考定价') OR TI=('药价改革') OR TI=('药品定价改革') OR KY=('医保支付') OR KY=('药品支付') OR KY=('药品参考价') OR KY=('药品参考定价') OR KY=('药价改革') OR KY=('药品定价改革') OR AB=('医保支付') OR AB=('药品支付') OR AB=('药品参考价') OR AB=('药品参考定价') OR AB=('药价改革') OR AB=('药品定价改革')) AND (FT=('重庆') OR FT=('绍兴') OR FT=('三明'))
Search 3	(SU=('医保支付') OR SU=('药品支付') OR SU=('药品参考价') OR SU=('药品参考定价') OR SU=('药价改革') OR SU=('药品定价改革') OR TI=('医保支付') OR TI=('药品支付') OR TI=('药品参考价') OR TI=('药品参考定价') OR TI=('药价改革') OR TI=('药品定价改革') OR KY=('医保支付') OR KY=('药品支付') OR KY=('药品参考价') OR KY=('药品参考定价') OR KY=('药价改革') OR KY=('药品定价改革') OR AB=('医保支付') OR AB=('药品支付') OR AB=('药品参考价') OR AB=('药品参考定价') OR AB=('药价改革') OR AB=('药品定价改革')) AND (TI=('中国') OR AB=('中国'))
**Wanfang Data:** http://www.wanfangdata.com.cn	
Search 1	主题:((医保支付+药品支付+药品参考价+药品参考定价+药价改革+药品定价改革)*(重庆+三明+绍兴+德国+日本+台湾+中国))+题名或关键词: ((医保支付+药品支付+药品参考价+药品参考定价+药价改革+药品定价改革)*(重庆+三明+绍兴+德国+日本+台湾+中国))+摘要: ((医保支付+药品支付+药品参考价+药品参考定价+药价改革+药品定价改革)*(重庆+三明+绍兴+德国+日本+台湾+中国))
**Weipu:** www.cqvip.com	
Search 1	(m=(医保支付+药品支付+药品参考价+药品参考定价+药价改革+药品定价改革)+r=(医保支付+药品支付+药品参考价+药品参考定价+药价改革+药品定价改革))*(m=(重庆+三明+绍兴+德国+日本+台湾+中国)+r=(重庆+三明+绍兴+德国+日本+台湾+中国))

Table S3. Profiles of interviewed key opinion leaders

Interviewee	Perspective	Profile
#1	Authorities	Leading position in the Institute of Social Security, the Ministry of Human Resources and Social Security and the China Health Insurance Research Association
#2	Authorities	Director of the Office of Urban and Rural Social Insurance Bureau in a mid-size city; initiated various pilot projects regarding drug reimbursement and pricing
#3	Authorities	Chief of the health insurance authority in one piloting city; in charge of the development of reimbursement and procurement lists and drug pricing
#4	Authorities	Manager of drug procurement authority in one piloting city; participated in the establishment of drug pricing policy in this city
#5	Authorities	Leading position in the provincial pricing authority which manages one piloting city; directly involved in drug pricing and related policy making
#6	Authorities	Academic representative; leading drug pricing related researches and other health policy-related research
#7	Prescribers	One of the Chinese leading physicians
#8	Prescribers	One of the leading physicians in one piloting city
#9	Prescribers	One of the leading physicians in one piloting city

Table S4. Drugs stopped or limited by the Shaoxing 7th People's Hospital (27)

Active ingredient; formulation	Brand name	Producer	Impact
Fluoxetine; capsules	Prozac	Patheon France	Stopped
Fluvoxamine; tablets	Luvox	Abbott Healthcare SAS France	Stopped
Lamotrigine; tablet	Lamictal	GSK Phamaceuticals S.A. Poland	Stopped
Mirtazapine; tablets	Remeron	N.V. Organon Netherlands	Stopped
Paliperidone; ER tablets	Invega	Janssen Cilag Manufacturing L.L.C Puerto Rico	Stopped
Tandospirone; capsules	LvKang	Keruide Sichuan China	Stopped
Ziprasidone; capsules	Zeldox	Pfizer Australia Pty Limited	Stopped
Cefoperazone/sulbactam; injections	Sulperazon	Pfizer	Limited use
Duloxetine; capsules	Cymbalta	Eli Lilly and Company USA	Limited use
Enteral nutrition emulsion	Fresubin Diabetes	Fresenius Kabi Germany	Limited use
Enteral nutrition suspension	能全力 (Nengquanli)	Nutricia Phamaceuticals Wuxi China	Limited use
Escitalopram; tablets	Lexapro	H. Lundbeck A/S Denmark	Limited use
Oxazepam; tablets	优菲 (Youfei)	Yimin Pharmaceuticals Beijing	Limited use
Venlafaxine; ER capsules	Effexor XR	Wyeth Medica Ireland	Limited use

ER – extended release

Table S5. Cities allowed to pilot the “2nd price negotiation” (29-31)

Area	First set of participating cities	Second set of participating cities	Third set of participating cities
Eastern	Anshan, Shanghai, Zhenjiang, Xiamen, Weifang, Shenzhen,	Tianjin, Shaoxing, Ningbo, Sanming, Zhuhai	Benxi, Nanjing, Wuxi, Xuzhou, Changzhou, Suzhou, Nantong, Lianyungang, Huaian, Yancheng, Yangzhou, Taizhou, Hangzhou, Wenzhou, Fuzhou, Putian, Quanzhou, Zhangzhou, Nanping, Longyan, Ningde, Weihai, Dongying, Dongguan
Middle	Qitaihe, Wuhu, Maanshan, Luoyang, Ezhou, Zhuzhou	Tangshan, Taiyuan, Changchun, Xinyu, Sanya	Handan, Yuncheng, Yanbian, Qiqihaer, Hefei, Bengbu, Huainan, Huaibei, Tongling, Anqing, Huangshan, Chuzhou, Fuyang, Suzhou, Liuan, Haozhou, Chizhou, Xuancheng, Yingtan, Jiaozuo, Puyang, Wuhan, Changsha, Danzhou
Western	Zunyi, Kunming, Baoji, xining	Chongqing, Nanchong, Qingyang, Yinchuan, Kelamayi, Liuzhou, Eerduosi	Huhehaote, Wuhai, Yulin, Panzhihua, Guangyuan, Guiyang, Yuxi, Yanan, Baiyin, Haidong, Haixi, Hainanzhou, Haibei, Huangnan, Guoluo, Yushu, Wuzhong, Tulufan
Other	Beijing	x	x

Table S6. Feedback on international policies from primary research

Approach	Rating (1, 2, 3)						
	P1	P2	P3	P4	P5	P6	Average
Setting internal reference price groups for drugs with the same active ingredient (i.e. the same INN)	1	NA	1	1	1	1	1.00
Setting internal reference price groups for drugs with different INNs but belonging to the same pharmacological class	3	3	3	x	3	3	3.00
Setting internal reference price groups for drugs with different INNs and belonging to different pharmacological classes but with similar therapeutic benefit in treating a disease	2	2	3	x	2	3	2.40
Setting reference price (i.e. reimbursement level) at the average price of drugs from the group	1	1	2	1	2	2	1.50
Setting reference price (i.e. reimbursement level) at the weighted average price of drugs from the group	2	3	1	3	3	1	2.17
Setting reference price (i.e. reimbursement level) at the cheapest drug from the group	2	2	1	3	1	3	2.00
Setting reference price (i.e. reimbursement level) at the fixed % below the price of the off-patent original drug	3	1	3	3	3	3	2.67
Using ERP to set prices of reimbursable drugs not included into the internal reference price group system	2	1	x	x	2	1	1.50
Application of free pricing for reimbursable drugs included into the internal reference price group system (with reference price defining the reimbursement level)	x	1	x	x	3	1	2.33
Using ERP to set prices of non-reimbursable drugs	x	3	x	x	x	1	2.00
Application of free pricing for non-reimbursable drugs	1	3	x	x	x	1	1.67

ERP – external reference pricing; IRP – internal reference pricing, P1…P6 – payer 1 to 6, NA – not applicable, x – no answer
1 – Most relevant and applicable to China
2 – Quite relevant and applicable to China
3 – Not relevant and not applicable to China
